# Branched-chain amino acids deficiency promotes diabetic cardiomyopathy by activating autophagy of cardiac fibroblasts

**DOI:** 10.7150/thno.102708

**Published:** 2024-10-28

**Authors:** Ze-Yu Zhou, Kai Song, Zhi-Yan Liu, Yu-Fan Ke, Yan Shi, Ke Cai, Rui Zhao, Xin Sun, Hui Tao, Jian-Yuan Zhao

**Affiliations:** 1 Institute for Developmental and Regenerative Cardiovascular Medicine, MOE-Shanghai Key Laboratory of Children's Environmental Health, Xinhua Hospital, Shanghai Jiao Tong University School of Medicine, Shanghai 200092, China.; 2 Department of Anesthesiology and Perioperative Medicine, Department of Cardiothoracic Surgery, The Second Affiliated Hospital of Anhui Medical University, Hefei 230601, China.; 3 International Human Phenome Institutes (Shanghai), Shanghai 200433, China.

**Keywords:** Branched-chain amino acid, Diabetic cardiomyopathy, Autophagy, Cardiac fibroblasts, Cardiac fibrosis

## Abstract

**Rationale:** More than half of the patients with type II diabetes mellitus (T2D) develop diabetic cardiomyopathy (DCM). Glycemic control alone cannot effectively prevent or alleviate DCM.

**Methods:** Herein, we concentrated on the variations in levels of metabolites between DCM and T2D patients without cardiomyopathy phenotype. In high-fat diet/low-dose streptozotocin-induced T2D and leptin receptor-deficient diabetic mouse models, we investigated the effect of altering branched-chain amino acids (BCAAs) levels on DCM.

**Results:** We discovered that the levels of plasma BCAAs are notably lower in 15 DCM patients compared to 19 T2D patients who do not exhibit cardiomyopathy phenotype, using nuclear magnetic resonance analysis. This finding was further validated in two additional batches of samples, 123 DCM patients and 129 T2D patients based on the BCAA assay kit, and 30 DCM patients and 30 T2D patients based on the LC-MS/MS method, respectively. Moreover, it is verified that BCAA deficiency aggravated, whereas BCAA supplementation alleviated cardiomyopathy phenotypes in diabetic mice. Furthermore, BCAA deficiency promoted cardiac fibroblast activation by stimulating autophagy in DCM mice. Mechanistically, BCAA deficiency activated autophagy via the AMPK-ULK1 signaling pathway in cardiac fibroblasts. Using pharmacological approaches, we validated our findings that autophagy inhibition relieved, whereas autophagy activation aggravated, DCM phenotypes.

**Conclusions:** Taken together, we describe a novel perspective wherein BCAA supplementation may serve as a potential therapeutic agent to mitigate DCM and fibrosis. Our findings provide insights for the development of preventive measures for DCM.

## Background

Cardiovascular disease is the leading complication of type II diabetes mellitus (T2D). Diabetic cardiomyopathy (DCM) is a major cause of T2D-associated mortality^1^, ^2^ with a prevalence of 20-60% in this population.^3^, ^4^ DCM lacks a standardized definition and generally refers to diabetes-related myocardial dysfunction in the absence of underlying structural heart disease, coronary artery disease, or hypertension.^5^, ^6^

Glycemic exposure has long been recognized as a major predictor of DCM risk in T2D.^7^-^9^ Despite strong epidemiological links between poor glycemic control and DCM risk, the effects of intensified glycemic control on DCM prevention remain controversial.^6^ In individuals with T2D, glycemic control alone cannot effectively prevent or alleviate DCM,^6^, ^8^ suggesting that factors other than glycemic metabolites contribute to DCM development. Metabolomics approaches have shown that metabolites such as glucose, fructose, amino acids, and lipids are typically altered in T2D.^10^ Because DCM has long been considered as a complication of T2D, the differences between DCM and T2D have been ignored. The diabetic heart is characterized by metabolic disturbances which, together with subcellular component abnormalities and immunological alterations, locally prompt inflammation, oxidative stress, mitochondrial dysfunction, and apoptosis.^11^ Understanding the metabolic differences between T2D and DCM is fundamental for revealing pathophysiological factors underlying DCM and developing new therapeutic targets.

One of the strongest associations between diabetes and glycemic traits that have emerged from metabolomics studies is the positive association with branched-chain amino acids (BCAAs), including leucine, isoleucine, and valine. BCAA levels are elevated in obese individuals and those with increased insulin resistance.^12^, ^13^

Autophagy is rapidly activated in response to nutrient and energy stresses, including inadequate nutrient supply and deprivation of growth factors.^14^ Previous studies reported that BCAA accumulation inhibits autophagy via the mTOR pathway, whereas conversely, reduced BCAA levels activate autophagy.[15, 16]A recent study demonstrated that the loss of autophagy impedes cancer-associated fibroblast activation and collagen production.^17^ Increasing evidence shows the essential role of autophagy in promoting the activation and proliferation of cardiac fibroblasts, which leads to myocardial fibrosis.^17^-^22^

Although BCAAs are considered important risk factors for diabetes, the current study found that BCAAs exhibited a protective effect against DCM. Decreased plasma BCAA levels in patients with T2D were associated with increased DCM risk. Mechanistically, BCAA deficiency promoted cardiac fibroblast activation by activating autophagy, whereas BCAA supplementation improved cardiac function and reduced the degree of cardiac fibrosis in DCM mice.

## Results

### Decreased Plasma BCAA Levels Are Associated With Diabetic Cardiomyopathy

To investigate whether the metabolite profiles of patients with T2D were associated with DCM risk, 50 patients were screened. Of these, 15 patients with DCM and 19 age- and sex-matched patients with diabetes as controls fulfilled all inclusion criteria. The clinical characteristics of patients with DCM and diabetic controls are presented in **Table S1**.

**Figure 1:**
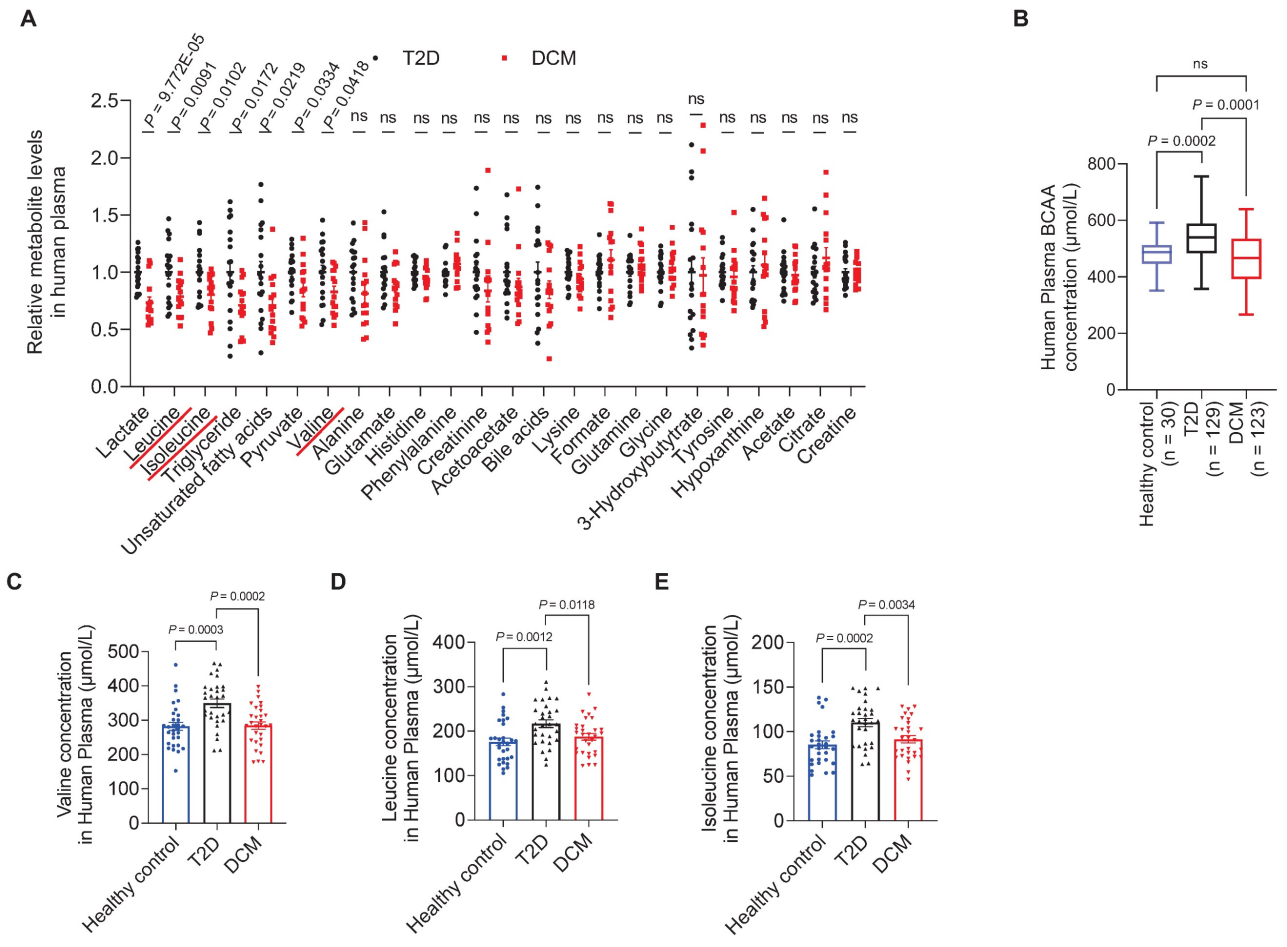
** Decreased plasma BCAA levels are associated with diabetic cardiomyopathy. A**, Relative plasma concentrations of metabolites in T2D controls and patients with DCM measured by NMR. **B**, Plasma concentrations of BCAAs in healthy controls, T2D, and DCM measured using a BCAA assay kit. **C-E**, Plasma concentrations of valine, leuvine and isoleucine in healthy controls, T2D, and DCM measured using LC-MS/MS. Data are expressed as mean±SEM. The nonparametric two-tailed Student's *t*-test was used to compare groups. Significance is indicated as ^ns^*P* > 0.05, *P < 0.05, **P < 0.01, ***P < 0.001, and ****P < 0.0001. See also Figure S1.

Fasting plasma samples were collected and metabolite profiling was performed using Nuclear Magnetic Resonance (NMR). Of the 24 successfully identified and quantified metabolites, 11 were common amino acids (**Figure S1**). When assessing the correlations between plasma metabolite concentrations and DCM occurrence, we found that the concentrations of BCAAs (leucine, isoleucine, and valine) were significantly associated with DCM. The levels of leucine, isoleucine, and valine in the DCM group were decreased by 21.34% (*P* = 0.009), 19.85% (*P* = 0.010), and 17.10% (*P* = 0.042), respectively, compared to the levels in the diabetic group (**Figure 1A**).

To validate these findings, we screened another 350 diabetic patients, of which 123 patients with DCM and 129 age- and sex-matched patients with diabetes fulfilled the inclusion criteria. Furthermore, 30 age- and sex-matched healthy individuals were recruited as controls (**Table S2**). According to BCAA assay results, the BCAA levels in the T2D group were higher than those in the healthy control group (*P* = 0.0002), whereas the BCAA levels exhibited a 15% decrease (*P* = 0.0001) in patients with DCM compared to T2D patients without cardiomyopathy (**Figure 1B**). Moreover, we screened a third cohort of diabetic patients (**Table S3**), in which plasma samples from 30 DCM patients and 30 T2D patients were measured for BCAA concentrations by LC-MS/MS. The levels of valine, leucine, and isoleucine in the DCM group were decreased by 18.65% (*P* = 0.0002), 13.58% (*P* = 0.0118), and 17.03% (*P* = 0.0034), respectively, compared to the levels in the T2D group (**Figure 1C-E**).

This suggests that although high BCAA levels are a risk factor for developing T2D, BCAAs exhibited protective effects in patients with DCM who had already developed T2D. These results from clinical samples indicate that decreased BCAA levels are associated with the presence of DCM.

The study design was approved and supervised by the Ethics Committee of Anhui Medical University, following the criteria established by the Declaration of Helsinki. The ethical approval number for medical research was 2021061. All participants or their authorized representatives provided written informed consent prior to study-related procedures.

### BCAA Deficiency Aggravates Cardiomyopathy Phenotypes in the Two Types of T2D Mouse Models

**Figure 2:**
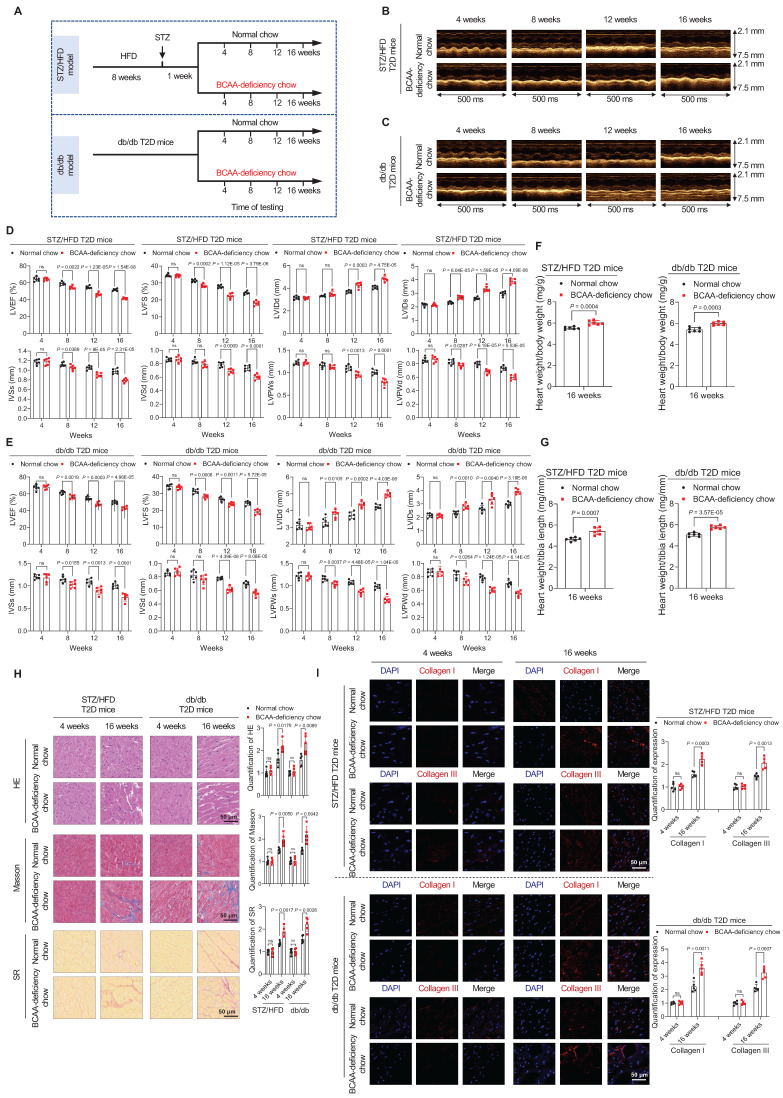
** BCAA deficiency time-dependently impairs cardiac function in STZ/HFD and db/db T2D mice. A**, Schematic representation of the establishment of the two T2D models. **B,C**, M-mode echocardiogram images of STZ/HFD T2D mice (**B**) and db/db T2D mice (**C**) at four-week intervals after BCAA-deficient chow feeding (n=6 mice in each group). **D,E**, Echocardiography analysis illustrating the worsened heart function in STZ/HFD T2D mice (**D**) and db/db T2D mice (**E**) after BCAA-deficient chow feeding (n=6 mice in each group). **F,G**, Ratios of heart weight to body weight (**F**) and heart weight to tibia length (**G**) in indicated groups after 16 weeks of BCAA-deficient chow feeding (n=6 mice in each group). **H**, Hematoxylin and eosin, Masson's trichome, and Sirius Red stainings of heart tissues in indicated groups after 16 weeks of BCAA-deficient chow feeding. The image quantification is shown on the right (n = 5 mice in each group). **I**, Increased levels of Collagen I and III as detected via immunostaining of heart tissues from STZ/HFD and db/db T2D mice after 16 weeks of BCAA-deficient chow feeding. The image quantification is shown on the right (n = 5 mice per group). Data are expressed as mean±SEM. The nonparametric two-tailed Student's *t*-test was used to compare groups. Significance is indicated as ^ns^*P* > 0.05, *P < 0.05, **P < 0.01, ***P < 0.001, and ****P < 0.0001. See also Figures S2 and S3.

Next, we explored whether decreased BCAA levels increase the risk of cardiomyopathy in both an STZ/HFD-induced T2D mouse model (STZ/HFD T2D mice) and a leptin receptor-deficient (db/db) mouse model of induced T2D. The process of generating these T2D models is shown in **Figure 2A**. In both mouse models, we observed T2D phenotypes, including hyperglycemia (**Figure S2A,B**) and insulin resistance, as detected by glucose tolerance tests (**Figure S2C,D**) and insulin tolerance tests (**Figure S2E,F**).

To study whether BCAA deficiency promotes the onset of cardiomyopathy in these T2D mouse models, standard or BCAA-deficient chow was administered to STZ/HFD and db/db T2D mice (**Figure 2A**). The ratio of BCAA to normal chow was optimized to ensure that the decreased circulating BCAA levels induced by BCAA-deficient chow were comparable to those observed in human patients. Compared to normal chow containing 12 g/kg leucine, 8 g/kg isoleucine, and 8 g/kg valine, BCAA-deficient chow contained 6 g/kg leucine, 4 g/kg isoleucine, and 4 g/kg valine. The mice fed with BCAA-deficient chow exhibited a 22% decrease in total blood BCAAs in the STZ/HFD model (**Figure S3A**) and a 21% decrease in the db/db mouse model, compared to mice fed with normal chow (**Figure S3B**).

Mice with T2D were fed either standard chow or BCAA-deficient chow for 16 weeks (**Figure 2A**). At this stage, BCAA-deficient chow did not alter food intake (**Figure S3C,D**), water intake (**Figure S3E,F**), fasting blood glucose levels (**Figure S3G,H**), and insulin resistance, as detected by glucose tolerance tests (**Figure S3I,J**) and insulin tolerance tests (**Figure S3K,L**) in both STZ/HFD and db/db mice. The BCAA-deficient chow reduced and the activity of mTOR and BCAA catabolic enzymes in STZ/HFD and db/db mouse hearts (**Figure S3M**).

In both STZ/HFD and db/db T2D mouse models, cardiac function was monitored using echocardiography at 4, 8, 12, and 16 weeks after changing standard to BCAA-deficient chow. Echocardiography results showed that the cardiac function of T2D mice gradually declined. However, T2D mice with BCAA deficiency showed a faster deterioration of cardiac function. From week 4 to 8 of BCAA deficiency, the cardiac function of these mice was worse than that of the mice on a normal diet. From week 12 to 16, cardiac function significantly deteriorated (**Figure 2B,C**) as evidenced by lower LVEF and LVFS, larger left ventricular internal dimension in end-systole and end-diastole (LVIDs and LVIDd, respectively), thinner interventricular septum in end-systole and end-diastole (IVSs and IVSd, respectively), and thinner left ventricular posterior wall in end-systole and end-diastole (LVPWs and LVPWd, respectively; **Figure 2D,E**). Additionally, increased heart weight/body weight and heart weight/tibia length ratios were detected in the BCAA-deficient chow-fed group after 16 weeks of feeding (**Figure 2F,G**). Together, these echocardiography results indicate that BCAA deficiency impairs cardiac function in a time-dependent manner in T2D mice. All animal experimental procedures were conducted in strict compliance with the NIH Guide for the Care and Use of Laboratory Animals (8th edition, 2011) and had received the necessary approval from the Institutional Animal Care and Use Committee of Anhui Medical University (approval number: LLSC 20218352).

Considering that impaired heart function is closely associated with cardiac fibrosis, we next examined fibrosis levels by assessing both histopathology and expression of protein markers of fibrosis. HE, Masson's trichrome, and SR stainings showed that BCAA-deficient chow caused severe cardiac fibrosis in STZ/HFD and db/db T2D mouse models after 16 weeks of feeding (**Figure 2H**). Furthermore, in immunofluorescence stainings, type I and type III collagen levels were significantly increased in heart tissues from T2D mice fed BCAA-deficient chow, compared to T2D mice fed normal chow (**Figure 2I**). Taken together, these results indicate that BCAA deficiency increases the cardiomyopathy risk in diabetes.

### BCAA Supplementation Alleviates Cardiomyopathy Phenotypes in T2D Mice

**Figure 3:**
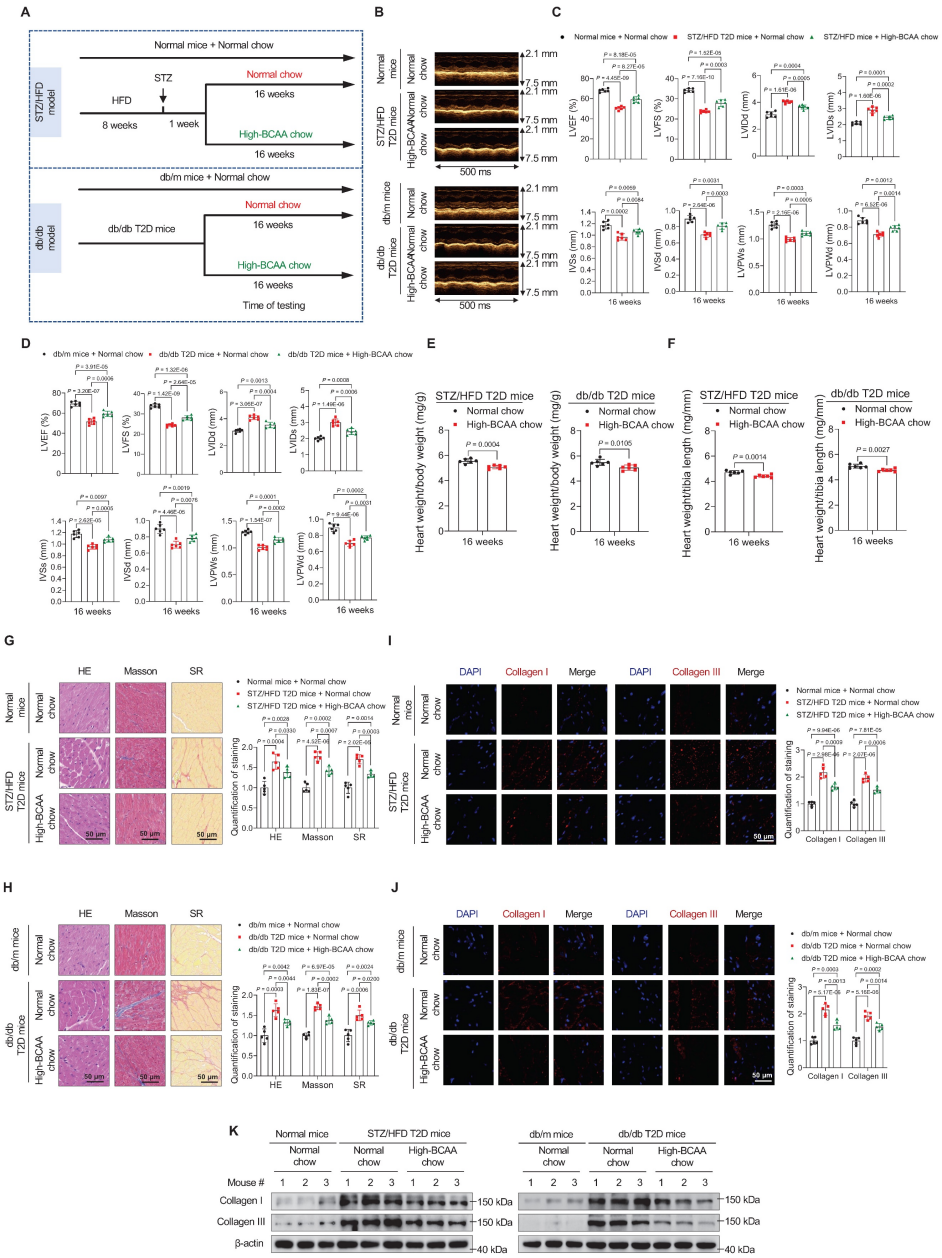
** BCAA supplementation in diabetes prevents the occurrence of cardiomyopathy. A**, Schematic representation of the establishment of mice models. **B**, M-mode echocardiogram images of indicated groups after 16 weeks of high-BCAA chow feeding (n = 6 mice in each group). **C,D**, Echocardiography analysis showing that high-BCAA chow feeding rescues cardiac function in STZ/HFD (**C**) and db/db (**D**) T2D mice (n = 6 mice in each group). **E,F**, Ratios of heart weight to body weight (**E**) and heart weight to tibia length (**F**) in indicated groups after 16 weeks of high-BCAA chow feeding (n=6 mice in each group). **G,H**, Hematoxylin and eosin, Masson's trichome, and Sirius Red stainings of heart tissues in indicated groups after 16 weeks of high-BCAA chow feeding. The image quantification is shown on the right (n = 5 mice in each group). **I,J**, Decreased levels of Collagen I and III as detected via immunostaining of heart tissues from STZ/HFD (**I**) and db/db (**J**) T2D mice after 16 weeks of high-BCAA chow feeding. The image quantification is shown on the right (n = 5 mice per group). **K**, Decreased levels of Collagen I and III in heart tissues from T2D mice fed with high-BCAA chow, as detected via western blotting (n = 5 mice in each group). Data are expressed as mean±SEM. The nonparametric two-tailed Student's *t*-test was used to compare groups. Significance is indicated as ^ns^*P* > 0.05, *P < 0.05, **P < 0.01, ***P < 0.001, and ****P < 0.0001. See also Figure S4.

Next, we investigated whether increased BCAA levels can prevent the occurrence of cardiomyopathy in T2D mice. Standard or high-BCAA chow was fed to STZ/HFD and db/db T2D mice (**Figure 3A**). Compared with normal chow containing 12 g/kg leucine, 8 g/kg isoleucine, and 8 g/kg valine, high-BCAA chow contained 24 g/kg leucine, 16 g/kg isoleucine, and 16 g/kg valine. High-BCAA chow induced a 39% increase in total BCAA levels in the blood of STZ/HFD T2D mice (**Figure S4A**) and a 37% increase in total BCAA levels in the blood of db/db T2D mice, compared to mice fed normal chow (**Figure S4B**). High-BCAA chow feeding did not alter food intake (**Figure S4C,D**), water intake (**Figure S4E,F**), fasting blood glucose levels (**Figure S4G,H**), and insulin resistance, as detected by glucose tolerance tests (**Figure S4I,J**) and insulin tolerance tests (**Figure S4K,L**) in both STZ/HFD and db/db mice, compared with normal chow-fed mice. The High-BCAA chow also increased the activity of mTORC1 and BCAA catabolic enzymes in STZ/HFD and db/db mouse hearts (**Figure S4M**).

Cardiac function was echocardiographically assessed after 16 weeks of feeding in the STZ/HFD and db/db groups. Although the cardiac function of T2D mice showed a declining trend, high-BCAA chow significantly rescued cardiac function (**Figure 3B**). Moreover, echocardiography assay showed that high-BCAA chow-fed diabetic mice exhibited improved cardiac function compared to standard chow-fed mice, including better indices of LVEF, LVFS, LVIDd, LVIDs, IVSs, IVSd, LVPWs, and LVPWd (**Figure 3C,D**). Moreover, decreased heart weight/body weight and heart weight/tibia length ratios were detected in the high-BCAA chow-fed group at 16 weeks (**Figure 3E,F**), suggesting that BCAA supplementation may reduce the risk of DCM development in mice with T2D.

HE, Masson's trichrome, and SR stainings showed that at 16 weeks after high-BCAA feeding, decreased cardiac fibrosis was observed compared to groups with normal feeding (**Figure 3G,H**). Moreover, type I and type III collagen levels were decreased in heart tissues from diabetic mice fed high-BCAA chow compared to diabetic mice fed normal chow, using both immunofluorescence (**Figure 3I,J**) and western blotting (**Figure 3K**). Collectively, these results indicate that augmentation of BCAA levels in diabetes prevents the occurrence of cardiomyopathy.

### BCAA Deficiency Promotes Cardiac Fibroblast Activation by Stimulating Autophagy in Mice With DCM

**Figure 4:**
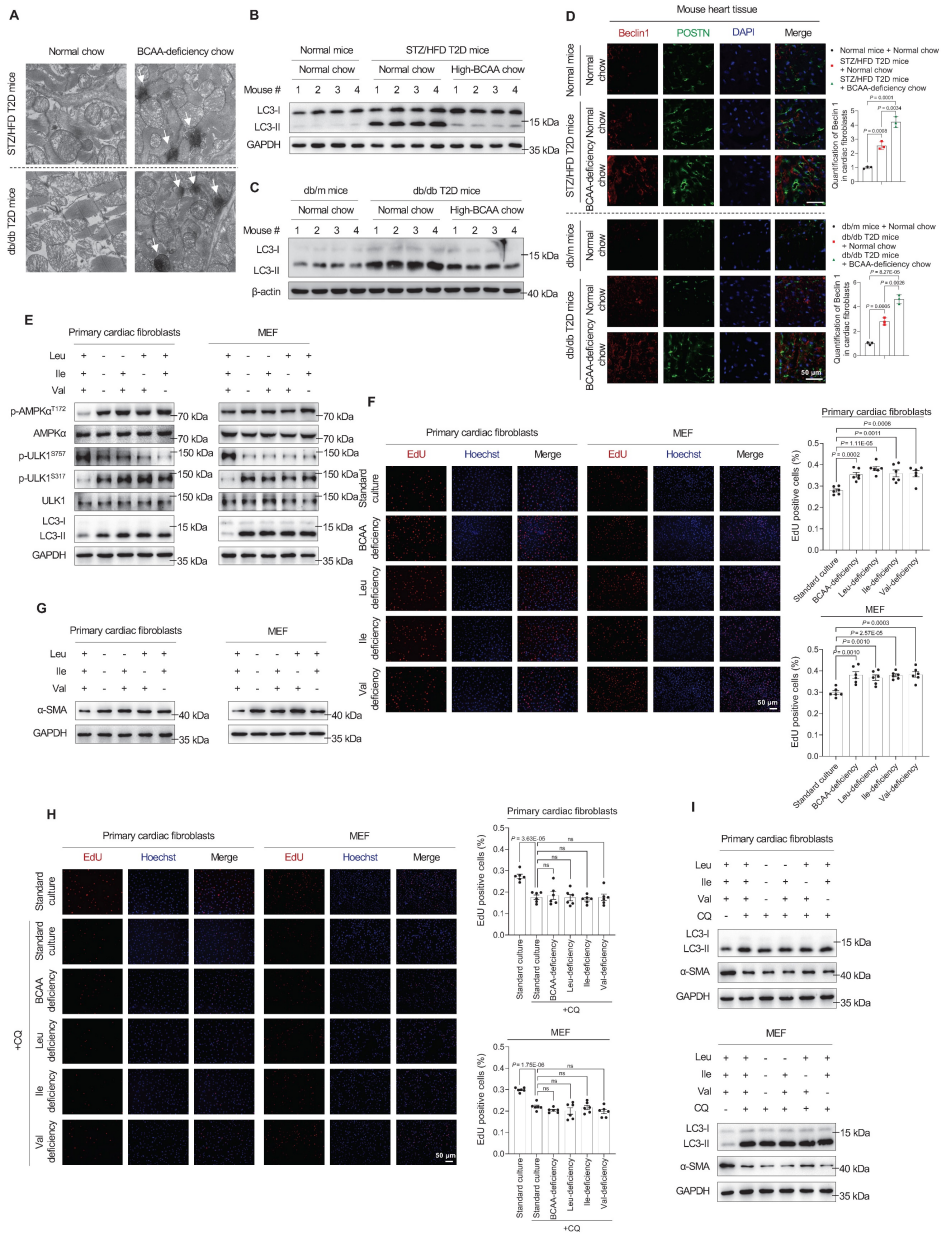
** BCAA deficiency promotes cardiac fibroblast activation by stimulating autophagy. A**, Autolysosomes detected by transmission electron microscopy in heart tissues of DCM mice (n = 6 mice in each group). **B,C**, Expression of the autophagy marker LC3-II in heart tissue samples from STZ/HFD (**B**) and db/db (**C**) T2D mice, detected via western blotting (n = 6 mice in each group). **D**, Strong colocalization of the autophagy marker Beclin 1 and POSTN proteins in heart tissues of DCM mice as detected by immunostaining. The image quantification is shown on the right (n = 3 mice in each group). **E**, Western blot analysis of AMPKα, p-AMPKα (T172), ULK1, p-ULK1 (S757 and S317), and LC3-II in primary cardiac fibroblasts and MEFs treated with BCAA deficiency (n = 3 in each group). **F**, EdU assay to detect the proliferation of primary cardiac fibroblasts and MEF treated with BCAA deficiency. The image quantification is shown on the right (n = 6 in each group). **G**, Western blot analysis of α-SMA expression in primary cardiac fibroblasts and MEF treated with BCAA deficiency (n = 3 in each group). **H**, EdU assay to detect the proliferation of primary cardiac fibroblasts and MEF treated with BCAA deficiency and chloroquine. The image quantification is shown on the right (n = 6 in each group). **I**, Western blot analysis of α-SMA expression in primary cardiac fibroblasts and MEF treated with BCAA deficiency and chloroquine (n = 3 in each group). Data are expressed as mean±SEM. The nonparametric two-tailed Student's *t*-test was used to compare groups. Significance is indicated as ^ns^*P* > 0.05, *P < 0.05, **P < 0.01, ***P < 0.001, and ****P < 0.0001.

Next, we investigated how altered BCAA levels contributed to DCM risk. We used transmission electron microscopy to examine the hearts of mice with BCAA-deficiency-induced DCM and found increased autolysosomes in both STZ/HFD and db/db mouse models (**Figure 4A**). Based on reports that autophagy plays important roles in the development of DCM and other types of cardiovascular diseases,^17^-^22^ we hypothesized that BCAA deficiency contributes to DCM risk by activating autophagy.

Remarkably, in heart tissues from the DCM group, the expression of the autophagy marker LC3-II increased in the two T2D mouse models, indicating autophagy activation (**Figure 4B,C**). However, in DCM mice fed high-BCAA chow, these changes were reversed (**Figure 4B,C**). These observations indicate that increased autophagy is correlated with DCM onset. Moreover, immunofluorescence analysis revealed strong colocalizations of the autophagy markers Beclin 1 and POSTN in fibrotic heart tissues from BCAA-deficiency-induced T2D mice compared to controls (**Figure 4D**), indicating that increased Beclin 1 expression in cardiac fibroblasts rather than cardiomyocytes enhanced their autophagy activity.

When sufficient nutrients are available, ULK1 at Ser757 is phosphorylated and activity is reduced^23^. Upon nutritional starvation, AMPK promotes autophagy to maintain amino acid levels through direct phosphorylation of ULK1 at multiple sites, including Ser317.^24^-^27^ Therefore, we hypothesized that BCAA deficiency activates autophagy via the AMPK-ULK1 signaling pathway in cardiac fibroblasts.

To test this hypothesis, western blotting results showed that respective or combined BCAA deficiency increased AMPK phosphorylation at T172 in primary mouse cardiac fibroblasts and MEF (**Figure 4E**). ULK1 is a downstream enzyme in the AMPK signaling pathway that initiates autophagosome formation, and its activation is crucial for autophagy initiation. Correspondingly, the phosphorylation of ULK1 at S317 was upregulated, whereas that at S757 was downregulated (**Figure 4E**). Likewise, a significant increase in the level of the autophagosome marker LC3-II was observed (**Figure 4E**). These results indicated that AMPK activates the autophagy regulatory complex, thus promoting autophagosome biogenesis in primary cardiac fibroblasts. Next, we determined whether BCAA deficiency promotes the proliferation of cardiac fibroblasts. Utilizing EdU staining to monitor DNA synthesis, we found that respective or combined BCAA deficiency increased DNA synthesis in primary mouse cardiac fibroblasts and MEF (**Figure 4F**). To explore whether BCAA deficiency promotes the activation of cardiac fibroblasts, we tracked the expression of alpha-smooth muscle actin (α-SMA). Western blotting analysis revealed a significant increase in α-SMA expression after single or combined BCAA deficiency in primary mouse cardiac fibroblasts and MEF (**Figure 4G**).

To confirm that the activation of primary cardiac fibroblasts by BCAA deficiency occurs through autophagy, we examined the phenotypes of cells after treatment with the autophagy inhibitor chloroquine, which inhibits lysosomal activity and degradation of LC3. The autophagy inhibitor chloroquine led to the accumulation of LC3-II proteins (**Figure 4I**), this indicates that autophagy is blocked at this step. As previously reported^19^, α-SMA protein expression decreased when autophagy was inhibited. Furthermore, BCAA deficiency did neither upregulate α-SMA protein expression (**Figure 4I**) nor DNA synthesis (**Figure 4H**). Collectively, these findings suggest that autophagy is essential for BCAA deficiency to activate and promote the proliferation of primary cardiac fibroblasts.

### Autophagy Inhibition Relieves, Whereas Autophagy Activation Aggravates, Diabetic Cardiomyopathy Phenotypes in T2D Mouse Models

**Figure 5:**
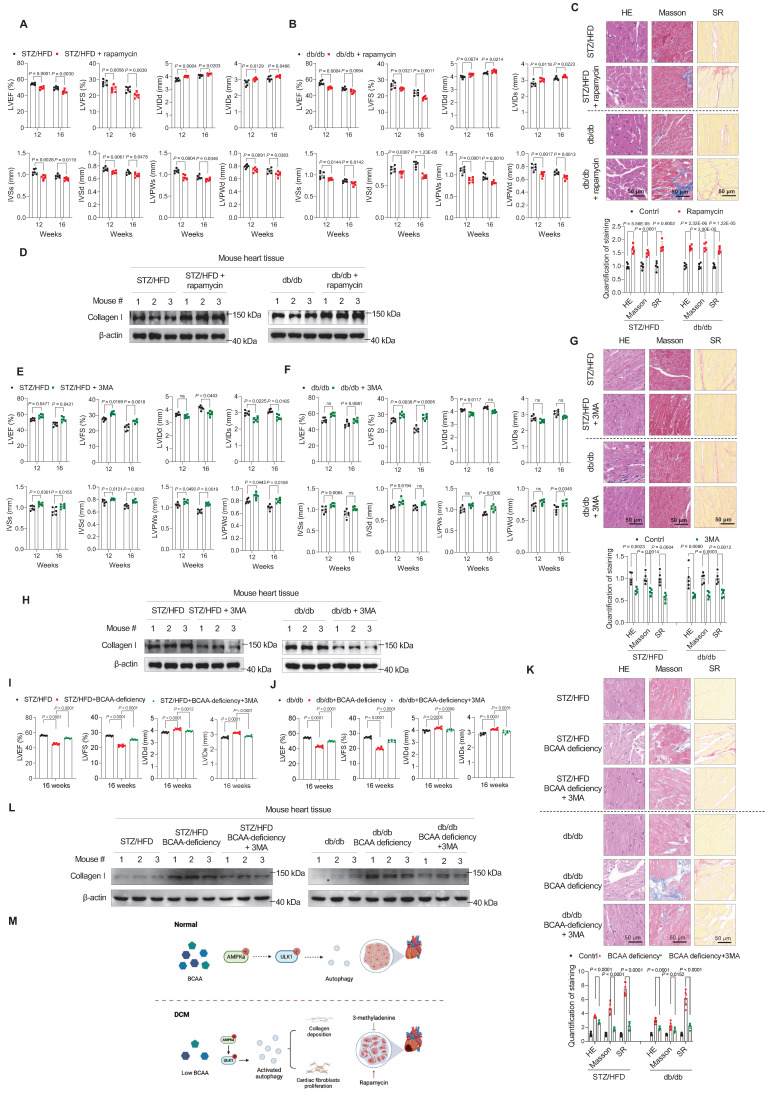
** Autophagy inhibition relieves, whereas autophagy activation aggravates, diabetic cardiomyopathy phenotypes. A,B**, Echocardiography analysis illustrating the worsened heart function in STZ/HFD (**A**) and db/db (**B**) T2D mice after gavage of rapamycin (n = 6 mice in each group). **C**, Hematoxylin and eosin, Masson's trichome, and Sirius Red staining of heart tissues in T2D mice after gavage of rapamycin. The image quantification is shown on the below (n = 5 mice in each group). **D**, Western blot analysis of Collagen I expression in T2D mice after gavage of rapamycin (n = 3 in each group). **E,F**, Echocardiography analysis illustrating the restored heart function in STZ/HFD (**E**) and db/db (**F**) T2D mice after gavage of 3-methyladenine (n = 6 mice in each group). **G**, Hematoxylin and eosin, Masson's trichome, and Sirius Red stainings of heart tissues in T2D mice after gavage of 3-methyladenine. The image quantification is shown on the below (n = 5 mice in each group). **H**, Western blot analysis of Collagen I expression in T2D mice after gavage of 3-methyladenine (n = 3 in each group). **I,J**, Echocardiography analysis illustrating the restored heart function in STZ/HFD (**I**) and db/db (**J**) BCAA-deficient mice after gavage of 3-methyladenine (n = 6 mice in each group). **K**, Hematoxylin and eosin, Masson's trichome, and Sirius Red stainings of heart tissues in BCAA-deficient mice after gavage of 3-methyladenine. The image quantification is shown on the below (n = 6 mice in each group). **L**, Western blot analysis of Collagen I expression in BCAA-deficient mice after gavage of 3-methyladenine (n = 3 in each group). **M**, Schematic showing that autophagy inhibition relieves, whereas autophagy activation aggravates, DCM phenotypes. Data are expressed as mean±SEM. The nonparametric two-tailed Student's *t*-test was used to compare groups. Significance is indicated as ^ns^*P* > 0.05, *P < 0.05, **P < 0.01, ***P < 0.001, and ****P < 0.0001.

To further confirm that altered autophagy is the key to the development of DCM in the two T2D mouse models, we tested whether targeting autophagy relieved or aggravated DCM. First, we tested whether autophagy activation promotes DCM onset. Therefore, we further activated autophagy in our two T2D mouse models by gavage administration of rapamycin (2 mg/kg/day). In our two T2D mouse models, 12 weeks and 16 weeks of the rapamycin treatment led to severe cardiac dysfunction, as evidenced by decreased LVEF, LVFS, IVSs, IVSd, LVPWs, and LVPWd and increased LVIDd and LVIDs (**Figure 5A,B**). According to HE, Masson, and SR stainings, cardiac fibrosis levels were increased in the rapamycin-treated group (**Figure 5C**). Furthermore, western blotting indicated increased protein levels of Collagen I in rapamycin-treated mice (**Figure 5D**). These results indicate that increased autophagy promotes DCM development in T2D mice.

Next, to mimic the inhibitory effect of BCAA in relieving DCM phenotypes, we used 3MA, a phosphoinositide 3-kinase inhibitor, to inhibit autophagy in the two mouse models. In the 3MA-treated group, 3MA (15 mg/kg/day by gavage) was additionally administered.

After 12 weeks of treatment, we found that compared to impaired cardiac function in DCM mice, the administration of 3MA restored the cardiac function of mice in both STZ/HFD and db/db models, as evidenced by the indices LVEF, LVFS, LVIDs, LVIDd, IVSs, IVSd, LVPWs, and LVPWd (**Figure 5E,F**). At 16 weeks, we confirmed the protective effect of 3MA against DCM development (**Figure 5E,F**). Moreover, cardiac fibrosis was assessed in the left ventricular myocardium using HE, Masson, and SR stainings. The results showed that 3MA treatment efficiently reduced cardiac fibrosis (**Figure 5G**). Western blotting also showed that Collagen I levels were decreased in 3MA-treated groups (**Figure 5H**).

Finally, to verify that BCAA deficiency promoted cardiac fibroblast activation by stimulating autophagy, we examined whether 3MA treatment could reduce fibrosis in BCAA-deficient mice. Compared to impaired cardiac function in BCAA-deficient mice, the administration of 3MA efficiently restored the cardiac function of mice (**Figure 5I,J**), and reduced cardiac fibrosis (**Figure 5K,L**) in both STZ/HFD and db/db models.

These results indicated that targeting autophagy prevented the development of DCM in T2D mice. Together with the finding that BCAA rescued the DCM phenotypes in mice (**Figure 3**), these findings indicate that autophagy inhibition relieved, whereas autophagy activation aggravated, DCM phenotypes (**Figure 5M**).

## Discussion

Here, we focused on the differences in metabolites between DCM and T2D and found that a decrease in BCAA levels was significantly associated with the occurrence of cardiomyopathy in patients with T2D. In both STZ/HFD- and db/db-induced T2D mouse models, BCAA-deficient chow feeding accelerated the development of DCM. In contrast, BCAA supplementation improved cardiac function and reduced the degree of cardiac fibrosis in DCM mice. Formal guidelines regarding DCM management have not been established; generally, therapy includes lifestyle modifications, improved glycemic control, lipid-lowering drugs, and the management of heart failure itself.^11^ Unfortunately, specific drugs for DCM treatment currently do not exist. We believe that many other meaningful metabolic differences waiting to be discovered to facilitate the early prevention and treatment of DCM.

BCAAs are essential amino acids and important nutrient signals that have both direct and indirect effects.^28^ BCAA supplementation and BCAA-rich diets are often associated with positive effects on body weight and muscle protein synthesis.^28^ However, the levels of circulating BCAAs tend to increase in obese individuals and are associated with worse metabolic health and future insulin resistance and T2D.^29^, ^30^ Recent examples of associations between BCAA and cardiometabolic disease phenotypes have raised the conjecture that circulating BCAAs are biomarkers, pathogenic agents, or both for these diseases.^30^-^36^ Nevertheless, the idea that BCAA supplementation directly contributes to the development of metabolic diseases remains controversial. Rats fed a low-fat diet supplemented with BCAAs do not develop insulin resistance.^30^ Several studies suggested that BCAA supplementation or BCAA-rich diets are beneficial for promoting lean body mass in obesity or catabolic disorders or for increasing satiety for body weight loss.^29^ A study on the synergistic effect of exercise and BCAAs on brain structure and function found that BCAA and exercise decreased body weight, decreased fasting insulin levels, and improved circadian rhythms. Only exercising mice fed dietary BCAAs showed improvements in spatial learning and memory.^37^ Similarly, BCAA deficiency in the brain triggers neurobehavioral alterations in mice.^38^ A study of diabetic hearts showed that leucine supplementation rescued the heart from lipid overload-induced insulin resistance and contractile dysfunction by targeting the endosomal mTOR-v-ATPase axis.^39^ Some studies have shown that BCAA deficiency is associated with certain diseases. BCAA insufficiency leads to premature ovarian insufficiency,^40^ and decreased serum BCAA levels have been observed in patients with chronic heart failure.^41^ A BCAA dysmetabolism model proposes that the accumulation of downstream metabolites (not BCAAs *per se*) promotes β-cell mitochondrial dysfunction, stress signaling, and apoptosis associated with T2D.^29^ Increased BCAA levels are more likely to mark the loss of insulin action than being causative. Increases in circulating BCAAs in obesity result, in part, from decreased rates of their oxidation in adipose tissue, due to coordinated transcriptional suppression of all BCAA catabolic enzymes, as well as from increased phosphorylation and inactivation of the branched-chain ketoacid dehydrogenase (BCKDH) complex in the liver, such that fewer BCAAs are taken up from the blood.^30^, ^42^ Overall, whether BCAAs or BCAA supplementation are harmful, beneficial, or neutral requires further investigation.

Our results indicate that BCAAs mediate the DCM risk by altering autophagy. The role of autophagy in cardiovascular diseases, including DCM, is controversial because autophagy can positively or negatively regulate myocardial diseases. The activation of autophagic responses may serve as a compensatory response to protect against cell death in the presence of insulin resistance because the degradation of unnecessary and unfavorable cellular components by autophagy is essential for maintaining normal cellular architecture and function.^14^ In preclinical studies, several pharmacotherapeutic agents attenuated DCM through autophagy-regulatory mechanisms by targeting specific or multiple effectors,^43^ including mTOR/AMPK, FOXO, and SIRT.^27^, ^44^-^48^ Some of these antidiabetic, antioxidant, and lipid-lowering agents exhibit cardioprotective effects in patients with diabetes and can simultaneously regulate cardiac autophagy in diabetic settings.^43^ However, the clinical success of specific autophagy regulators against DCM remains to be achieved. The cardiac autophagy status is a key factor in selecting a therapeutic agent, and the interplay between signaling events in autophagy-regulatory mechanisms needs to be completely understood.

Our study has several limitations. First, the small clinical sample size may have limited the statistical power of the analysis. However, we verified the observations in mice and found that BCAA supplementation significantly improved cardiac function and fibrosis in DCM mice. Second, plasma BCAA concentrations were significantly lower in patients with DCM than in those with T2D, but the potential reasons were not explored. This phenomenon may be due to dietary habits, abnormal BCAA metabolism in other tissues, such as the liver, or both. Third, we only performed relative rather than absolute quantification of serum metabolites, which increases the difficulty of clinical referencing.

In summary, our study identified the adverse effects of BCAA deficiency and autophagy activation on the development of DCM and cardiac fibrosis. Using metabolomic methods to reveal metabolic changes during DCM development, dietary regulation has become a promising prevention and intervention strategy for DCM. Overall, our study provides unique insights into the metabolic differences between DCM and T2D, as well as the role of BCAAs in DCM management.

## Methods

### Study Participants

The study design was approved and supervised by the Ethics Committee of Anhui Medical University, following the criteria established by the Declaration of Helsinki. The ethical approval number for medical research was 2021061. All participants or their authorized representatives provided written informed consent prior to study-related procedures. The research involved adult participants with or without T2D. Fasting plasma samples were obtained from participants in the morning at the Second Affiliated Hospital of Anhui Medical University between December 2020 and September 2023.

In the discovery group, 50 patients were screened. Of these, 15 patients with DCM and 19 age- and sex-matched patients with diabetes as controls fulfilled all inclusion criteria. In the first validation group, an additional 350 diabetic patients were screened. Among them, 123 patients with DCM and 129 age- and sex-matched patients with diabetes fulfilled the inclusion criteria. In another validation group, 30 patients with DCM and 30 age- and sex-matched patients with diabetes fulfilled the inclusion criteria. Individuals with a history of cardiovascular disease, sickle cell disease, or metabolic disorders, including uncontrolled hypertension or hyperthyroidism, were excluded, as were participants with abnormal liver or kidney function. The inclusion criteria for patients with DCM were: 1) confirmed diabetes diagnosis; 2) evidence of systolic or at least moderate diastolic dysfunction after diabetes diagnosis; 3) no prior history of congenital heart, valve, or coronary artery disease or heart failure; and 4) well-controlled blood glucose levels. Age- and sex-matched individuals with diabetes but without systolic/diastolic dysfunction were included in the T2D group. Propensity score method was performed to match the confounders between T2D and DCM groups. The demographic characteristics of the discovery group are shown in **Table S1**. The demographic characteristics of the validation population are shown in **Table S2** and **Table S3**.

### Nuclear Magnetic Resonance Analysis of Metabolites

Nuclear magnetic resonance (NMR) analysis was adapted from published methods.^49^, ^50^ Metabolites from 200 μL plasma were extracted by vortexing with 400 μL phosphate buffer (0.045 M, pH 7.4). The resulting solution was centrifuged at 16,000×*g* (4°C) for 10 min before 570 μL supernatant was transferred into NMR tubes for analysis. All one-dimensional ^1^H NMR spectra were acquired at 298 K using a Bruker Advance III 600 MHz NMR spectrometer (600.13 MHz for proton frequency) equipped with an inverse cryogenic probe (Bruker Biospin, Karlsruhe, Germany) using a t_2_-edited spectrum with a standard Carr-Purcell-Meibom-Gill (CPMG) sequence (RD-90°-(τ-180°-τ)n-acq). In total, 64 transients were collected from 32k data points with a spectral width of 20 ppm for each sample. A series of 2D NMR spectra were acquired for signal assignment purposes including COSY, TOCSY, JRES, HSQC, HMBC. NMR spectra were processed using the V3.6.0 software package TOPSPIN (Bruker Biospin). For ^1^H NMR spectra, an exponential window function was employed with a line broadening factor of 1 Hz and zero-Hz-filled to 128 K before Fourier transformation. Each spectrum was then phase and baseline-corrected with the chemical shift referenced to the anomeric proton signal of α-glucose (δ 5.23). The spectrum of each sample was then integrated into bins with the width of 0.002 ppm (1.2 Hz) using AMIX software package (V3.8.3, Bruker Biospin). The relative content of plasma metabolites was calculated by the characteristic and least-overlapping NMR signals and normalized to the sample volume.

### LC-MS/MS Analysis for BCAAs

BCAAs concentrations were measured in serum using a quantitative ultra-performance liquid chromatography-tandem mass spectrometry (UPLC-MS/MS) platform (ACQUITY UPLC-Xevo TQ-S; Waters Corp. Milford, MA, USA). All chromatographic separations were performed with a Cortex UPLC C18 Column VanGuard pre-column (1.6 × 5 mm) and analytical column (2.1 × 50 mm). The elution solvents were water with 0.1% formic acid (A) and acetonitrile with 0.1% formic acid (B). The flow rate was 500 μL/min with the following mobile phase gradient: 0-0.5 min (1% B), 0.5-2 min (1%-10% B), 2-2.5 min (10%-15% B), 2.5-3 min (15% B), 3-4 min (15%-20% B), 4-4.5 min (20%-99%B), 4.5-5 min (99% B), 5-5.2 min (99%-1% B), 5.2-7 min (99% B). The cone and collision energy for each amino acid used the optimized settings from QuanOptimize application manager. All BCAA standards were obtained from Sigma-Aldrich (St. Louis, MO, USA). The stock solution of individual amino acids was mixed and prepared to obtain a series of amino acid calibrators. The raw data generated by UPLC-MS/MS were then processed using the MassLynx (v4.1, Waters, Milford, MA, USA) and the QuanMET software (v1.0, Metabo-Profile, Shanghai, China) to perform peak integration, calibration, and quantitation for each amino acid.

### BCAA Assay Kit

Total BCAA levels were measured using Branched Chain Amino Acid Assay Kit / BCAA Assay Kit (ab83374), following the assay protocol. Briefly, 20 μL human plasma was added to a 96-well plate, then BCAA assay buffer (30 μL/well) was added. Per well containing leucine standard or test samples, 50 μL reaction mix containing 46 μL assay buffer, 2 μL enzyme mix, and 2 μL developer solution 3/substrate mix was added and mixed well. After incubation in the dark for 30 min at room temperature, the optical density was measured at 450 nm using a microplate reader. Data were background-corrected, and standard curves were plotted for each sample to calculate BCAA concentrations in the samples.

### Animal Models

All animal procedures were performed in accordance with the Animal Care Committee guidelines at Anhui Medical University. The 8-week-old male C57BL/6 mice were obtained from Hangzhou Ziyuan Laboratory Animal Technology Co., Ltd. (Zhejiang, China), housed in polycarbonate cages, and provided free access to food and water under a 12 h light-dark cycle.

For the streptozotocin (STZ)/high-fat diet (HFD)-induced T2D model, mice were divided into experimental and control groups. The control group was fed a regular diet (10 *kcal*% fat, D12450J, Wuxi Fanbo Biotechnology Co., Ltd, China). To induce insulin resistance and obesity, the experimental group was fed an HFD (60 *kcal*% fat, D12492, same as above) for 8 weeks and received intraperitoneal injection of STZ 80 mg/kg (MS1601, Shanghai Maokang Biotechnology Co., Ltd, China). Leptin receptor-deficient (db/db) mice were used in a second experimental group, whereas db/m mice were used as the corresponding control group. After the T2DM models were successfully established, the mice were fed with BCAA-deficient or high-BCAA chow. All mice were fed an L-Amino Acids Rodent Diet (FB-A10021B, including isoleucine 8 g/kg, leucine 12 g/kg, valine 8 g/kg, same as above) for adaptation one week in advance. The control group continued to be fed the same. The BCAA-deficient group was fed a diet with halved BCAA (FB-A10022, including isoleucine 4 g/kg, leucine 6 g/kg, valine 4 g/kg, same as above) to ensure that their BCAA intake was lower than that of the control group. The high-BCAA group was fed a diet with double BCAA content (FB-A10023, including isoleucine 16 g/kg, leucine 24 g/kg, valine 16 g/kg, same as above) to ensure that their BCAA intake was higher than that of the control group^51^, ^52^. The detailed information of the macronutrient and amino acid composition of the diets is shown in **Table S4**. During the induction period of 16 weeks, food intake, water intake, blood glucose levels, and insulin resistance of mice were regularly measured to monitor T2D progression. Mice treated with 3-methyladenine (3MA) or rapamycin were divided into three groups: control, 3MA, and rapamycin. All groups received a standard feed. Mice of the 3MA and rapamycin treatment groups underwent gavage administration with 3MA or rapamycin at the appropriate time points. All mice were anaesthetised with 2% isoflurane gas (spontaneous inhalation) and euthanized by cervical dislocation. Blood serum and mouse tissues were collected at specific time points during the experiment.

All experimental procedures were conducted in strict compliance with the NIH Guide for the Care and Use of Laboratory Animals (8th edition, 2011) and had received the necessary approval from the Institutional Animal Care and Use Committee of Anhui Medical University (approval number: LLSC 20218352).

### Isolation and Culture of Primary Mouse Cardiac and Mouse Embryonic Fibroblasts

Primary mouse cardiac fibroblasts were isolated as described previously.^17^ In brief, the hearts of C57BL/6 suckling mice aged 1-3 days were harvested and cut into 1 mm pieces that were then digested with 0.25% trypsin for 10 min at 37°C. The separated cells, collected from the supernatant centrifuged at 1,500 rpm for 5 min, were cultured in Dulbecco's modified Eagle's medium (DMEM) supplemented with 10% fetal bovine serum (FBS, both Gibco), 100 U/mL penicillin, and 100 mg/mL streptomycin (both Invitrogen) for 1.5 h. After the adhesion of fibroblasts, non-adherent cardiomyocytes were removed. For mouse embryonic fibroblasts (MEF) cultures, uteri were isolated from 13.5-day pregnant mice. The head, limbs, and visceral tissues were removed from isolated embryos. The remaining bodies were digested, and the separated MEF were collected. Primary fibroblasts and MEF were cultured in DMEM supplemented with 10% FBS, 100 U/mL penicillin, and 100 mg/mL streptomycin at 37°C in humidified air with 5% CO_2_/95% O_2_ and used for experiments within three passages.

### Cell Culture and Treatment

For BCAA-deficiency experiments, cells were cultured in a custom-made BCAA-deprivation medium (Shanghai Zongji Biotechnology, Inc.) with the remaining components and concentrations identical to those in the abovementioned DMEM. Leucine-deficient medium was obtained by adding isoleucine and valine, and complete medium was obtained by adding all three BCAAs. To study BCAA-deficiency effects, cells at the logarithmic growth stage were seeded into 6-well plates for 6 h. For chloroquine treatment, cells were pretreated with 50 µM chloroquine for 12 h.

### Echocardiography

Before heart dissection, cardiac hemodynamics were ultrasonographically assessed using the ultrasound imaging system VINNO 6 (frequency 23 MHz, Vinno Corporation, Suzhou, China). Mice under 2% isoflurane inhalation anesthesia (Once every 4 weeks at most) underwent transthoracic echocardiography after the heart rate had been stabilized within the range of 450-500 beats/min. M-mode tracings were recorded in the long-axis view, with a minimum of six cardiac cycles analyzed to calculate various parameters, including left ventricular ejection fraction, left ventricular fractional shortening, left ventricular end-systolic diameter, and left ventricular end-diastolic diameter. Echocardiography was performed before opening the chest (baseline) and during the course of the experiment in all groups.

### Histopathological Staining

Heart tissues were fixed in 4% paraformaldehyde, embedded in paraffin, cut into 5 μm-thick sections, and placed on glass slides. Tissue sections were subjected to hematoxylin and eosin (HE), Sirius Red (SR, collagen detection), and Masson's trichrome (fibrosis detection) stainings according to standard procedures. Six microscopy images per heart sample were captured for each staining technique. Quantification of the fibrotic area was performed with ImageJ. For analysis of the collagen volume fraction (CVF), the collagen and total areas were quantified with ImageJ, and CVF was calculated as follows: CVF = collagen area / total area.

### Immunofluorescence Staining

Frozen tissue sections or cells on slides were initially fixed by using a 4% paraformaldehyde solution and subsequently permeabilized with 0.3% Triton X-100. To minimize non-specific binding, FBS was used for blocking. Subsequently, the samples were incubated overnight at 4°C overnight with primary antibodies against Collagen I (14695-1-AP; Proteintech), Collagen III (22734-1-AP; Proteintech), Beclin (ab207612; abcam), and POSTN (66491-1-Ig; Proteintech). Subsequently, they were probed with appropriate Alexa Fluor dye-conjugated secondary antibodies (1:200, 1 h at room temperature). Nuclei were counterstained with DAPI. Confocal fluorescence images were analyzed using Image J.

### 5-Ethynyl-2′-deoxyuridine Staining

Cell proliferation was tested using the BeyoClick™ EdU Cell Proliferation Kit with Alexa Fluor 555 (C0075S, Beyotime, China). For 5-ethynyl-2-deoxyuridine EdU assays, logarithmic growth stage cells were seeded in 6-well plate with 10 μM EdU reagent for 2 h. Cells were washed with PBS for 5 min twice, before incubating with 4% Paraformaldehyde for 15 min. After washing with PBS for 5 min twice, samples were permeated with 0.3% TritonX-100 in PBS, and dyed with reaction solution (C0075S, Beyotime, China). The procedure was performed according to the manufacturer's instructions. Finally, Hoechst 33342 was used to stain cell nuclei. EdU and Hoechst 33342 stainings were observed using a fluorescence microscope (Nikon, Tokyo, Japan). Image J (National Institutes of Health) was used to calculate the percentage of EdU-positive cells.

### Transmission Electron Microscopy

Heart tissue samples were removed from a 2.5% glutaraldehyde solution and divided into small 0.1 cm^3^ blocks. These tissue blocks were fixed with a 1% osmic acid solution and subsequently dehydrated using various ethanol concentrations. Finally, the tissue blocks were embedded in a mixture of acetone and embedding solution. These samples were subjected to a temperature-controlled solidification process, including overnight incubation at 37°C, followed by 12 h at 45°C, and finally 24 h at 60°C. Once solidified, the samples were cut into thin sections (thicknesses, 50-70 nm), stained, and observed using a transmission electron microscope (Hitachi, Tokyo, Japan). The images were analyzed using Image J to evaluate the number, size, and structure of the mitochondria.

### Western Blotting

Heart tissues or cultured cells were homogenized and lysed with 0.5% NP-40 buffer (50 mM Tris-HCl, pH 7.5; 150 mM NaCl; 0.5% Nonidet P-40) containing a protease inhibitor mixture (Sigma-Aldrich) for 30 min on ice. Lysates were centrifuged at 15,000×*g* for 20 min, and the supernatants were used as whole-cell extracts. Protein samples were subjected to western blotting, according to standard procedures. Detection was performed by measuring chemiluminescence using an ECL Plus Western Blotting Detection System on a Typhoon FLA 9500 (both GE Healthcare). The following primary anti- bodies were used for western blot analysis: anti-Collagen I (1:1000; 14695-1-AP; Proteintech), anti-Collagen III (1:1000; 22734-1-AP; Proteintech), anti-LC3-II (1:1000; #2775; CST), anti-AMPKα (1:1000; #5831; CST), anti-Phospho-AMPKα (Thr172) (1:1000; #2535; CST), anti-ULK1 (1:1000; #8054; CST), anti-Phospho-ULK1 (Ser757) (1:1000; #14202; CST), anti-Phospho-ULK1 (Ser317) (1:1000; #37762; CST), anti-GAPDH (1:1000; #2118; CST), anti-α-SMA (1:1000; ab7817; Abcam), anti-Phospho-mTOR (Ser2448) (1:10000; 80596-1-RR; Proteintech), anti-mTOR (1:5000; A2445; Abclonal), anti-Phospho-p70 S6 Kinase 1 (T389) (1:1000; AP1059; Abclonal), anti-p70 S6 Kinase 1 (1: 1000; A2190; Abclonal), anti-Phospho-BCKDHA (S293) (1:1000; ab302504; Abcam), anti-BCKDHA (1:5000; 30028-1-AP; Proteintech), anti-BCAT2 (1: 1000; 16417-1-AP; Proteintech), anti-β-actin (1:1000; 60004-1-Ig; Proteintech).

### Quantification and Statistical Analysis

Two-tailed Student's *t*-tests were used to analyze the association between NMR metabolites among different groups. Pooled results were expressed as the mean±SEM for other parameters, and two-tailed Student's *t*-tests were used for comparisons between groups. *P*<0.05 was considered statistically significant. Statistical analyses were performed using Prism software (version 8.0; GraphPad Software, Inc.) and Excel (Microsoft Corp.).

## Supplementary Material

Supplementary figures and tables.

## Abbreviations

3MA: 3-methyladenine
α-SMA: alpha-smooth muscle actin
BCAA: branched-chain amino acid
CVF: collagen volume fraction
DCM: diabetic cardiomyopathy
DM: diabetes mellitus
DMEM: Dulbecco's modified Eagle's medium
EdU: 5-ethynyl-2′-deoxyuridine
FBS: fetal bovine serum
HE: hematoxylin and eosin
HFD: high-fat diet
IVSd: interventricular septum in end-diastole
IVSs: interventricular septum in end-systole
LVEF: left ventricular ejection fraction
LVFS: left ventricular fractional shortening
LVIDd: left ventricular internal dimension in end-diastole
LVIDs: left ventricular internal dimension in end-systole
LVPWd: left ventricular posterior wall in end-diastole
LVPWs: left ventricular posterior wall in end-systole
MEF: mouse embryonic fibroblasts
NMR: nuclear magnetic resonance
SR: Sirius Red
STZ: streptozotocin
T2D: type II diabetes mellitus
