# Supplementary material for: Branched-chain amino acids deficiency promotes diabetic cardiomyopathy by activating autophagy of cardiac fibroblasts
Source: Theranostics. 2024 Oct 28;14(19):7333–48. doi: 10.7150/thno.102708 (PMC11626946; doi:10.7150/thno.102708)
Supplement: Supplementary file 1 — Supplementary figures and tables. [file thnov14p7333s1.pdf]

Figure S1

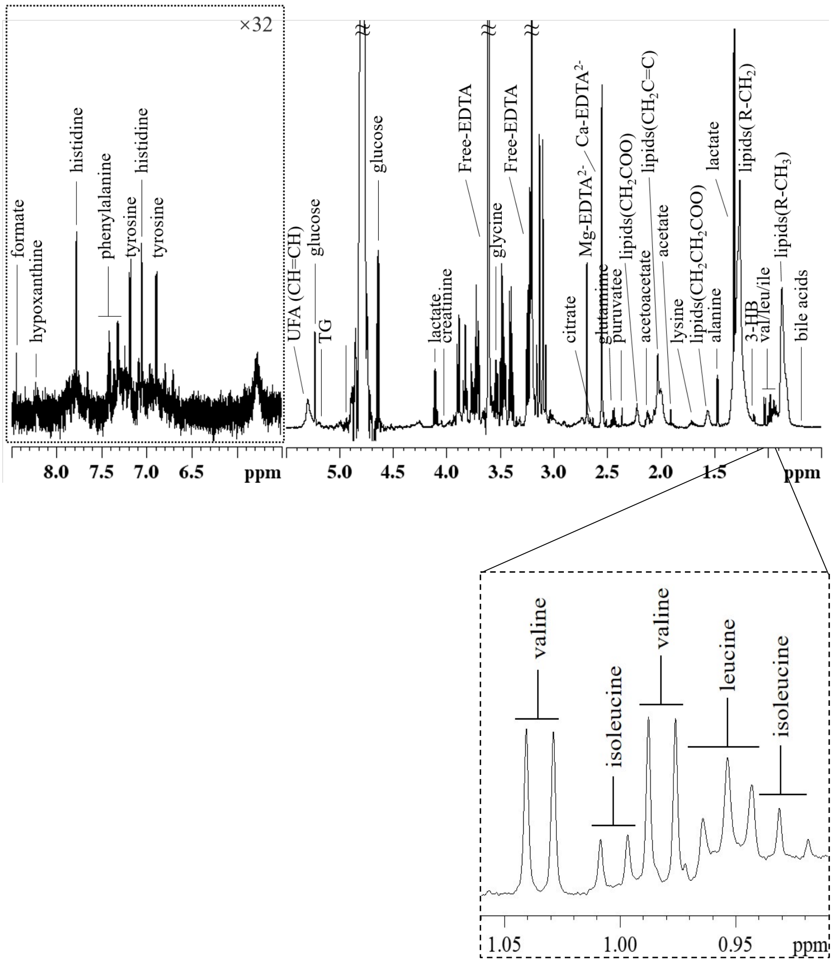

Figure S1. Metabolite profiling using nuclear magnetic resonance (NMR). Related to Figure 1.

Figure S2

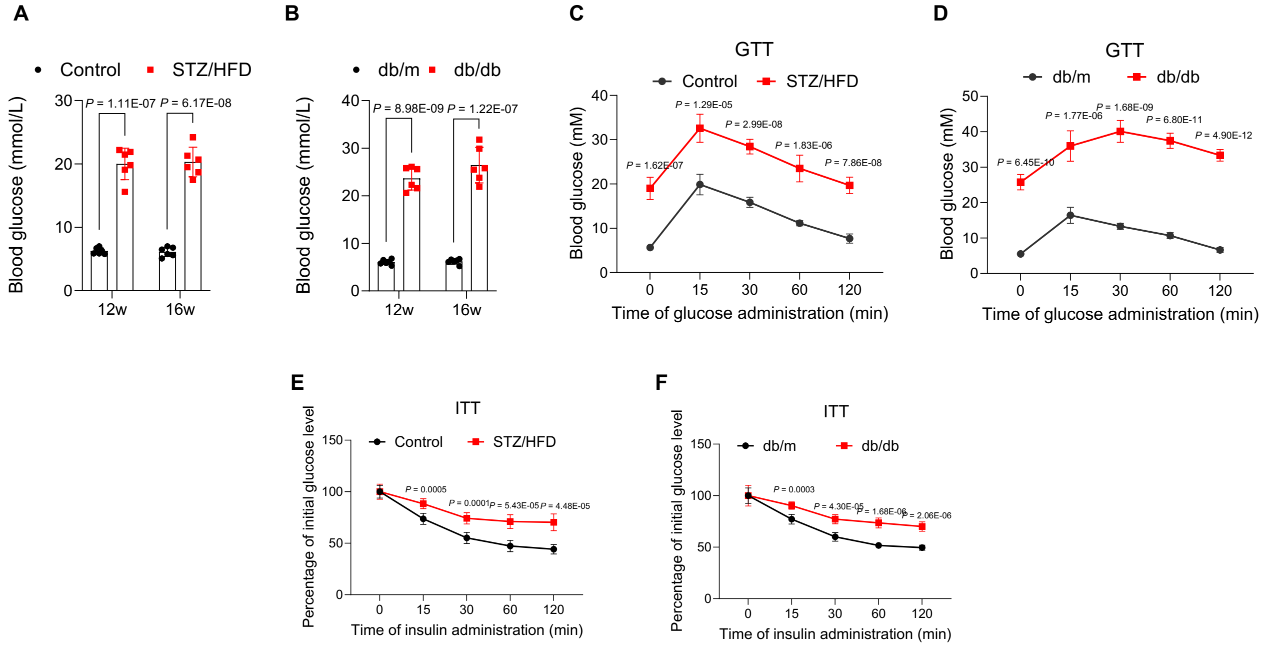

Figure S2. Information from STZ/HFD mice and db/db mice. Related to Figure 2.

A-B, Blood glucose of STZ/HFD T2D mice (A) and db/db T2D mice (B) in indicated groups (n = 6 mice in each group). C, D, Glucose tolerance were measured in fasted STZ/HFD T2D mice (C) and db/db (D) mice after 16 weeks of feeding (n = 6 mice in each group). E, F, Insulin tolerance were measured in fasted STZ/HFD T2D mice (E) and db/db T2D mice (F) after 16 weeks of feeding (n = 6 mice in each group). Data shown are expressed as mean ± SEM. $^{ns}P > 0.05$, $^{*}P < 0.05$, $^{***}P < 0.001$, using Student's *t*-test.

Figure S3

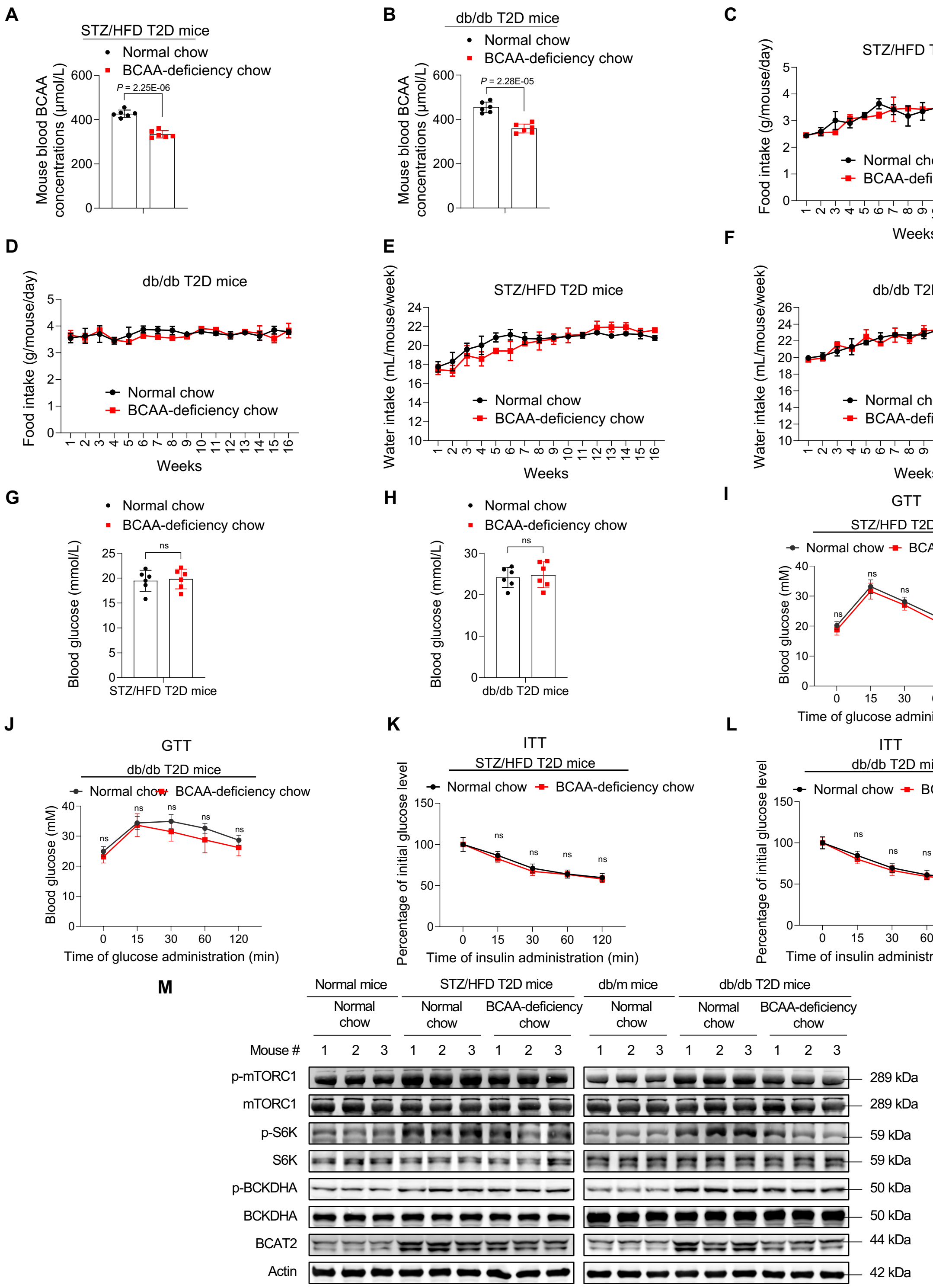

Figure S3. Information from T2D mice for either standard chow or BCAA-deficiency chow. Related to Figure 2.

A,B, Plasma concentration of BCAA in fasted STZ/HFD T2D mice (A) and db/db T2D mice (B) after 16 weeks of BCAA-deficiency feeding (n = 6 mice in each group). C,D, Food intake of STZ/HFD T2D mice (C) and db/db T2D mice (D) in indicated groups (n = 6 mice in each group). E,F, Water intake of STZ/HFD T2D mice (E) and db/db T2D mice (F) in indicated groups (n = 6 mice in each group). G,H, Blood glucose of STZ/HFD T2D mice (G) and db/db T2D mice (H) after 16 weeks of BCAA-deficiency feeding (n = 6 mice in each group). I,J, Glucose tolerance were measured in STZ/HFD T2D mice (I) and db/db T2D mice (J) in indicated groups (n = 6 mice in each group). K,L, Insulin tolerance were measured in fasted STZ/HFD T2D mice (K) and db/db T2D mice (L) in indicated groups (n = 6 mice in each group). M, Western blot analysis of mTORC1 pathway and BCAA metabolic enzymes in heart tissue samples from STZ/HFD and db/db T2D mice treated with BCAA-deficiency chow (n = 3 mice in each group). Data shown are expressed as mean ± SEM. $^{ns}P > 0.05$, $^{*}P < 0.05$, $^{***}P < 0.001$, using Student's *t*-test.

Figure S4

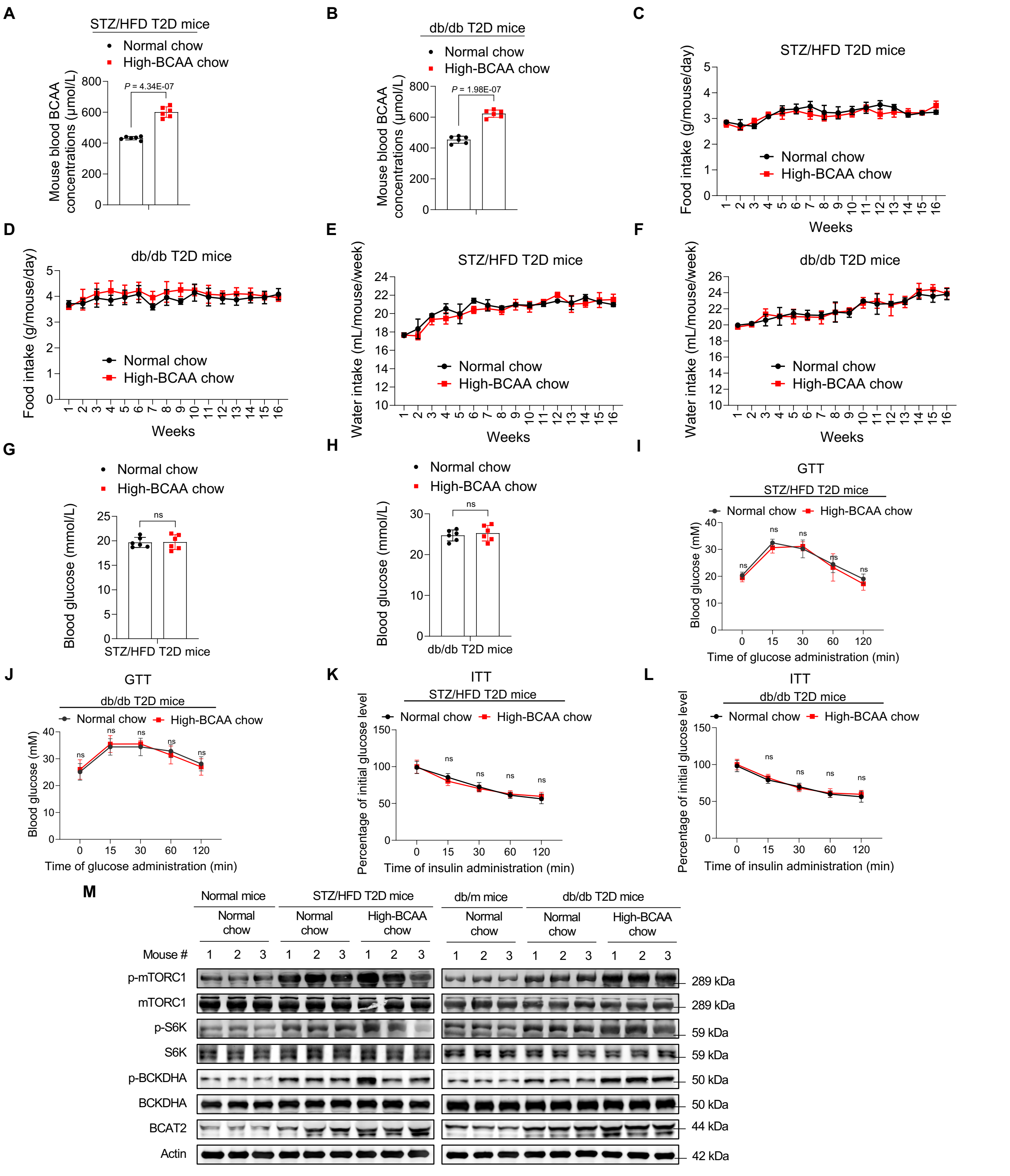

Figure S4. Information from T2D mice for either standard chow or High-BCAA chow. Related to Figure 3.

A,B, Plasma concentration of BCAA in faeted STZ/HFD T2D mice (A) and db/db T2D mice (B) after 16 weeks of High-BCAA feeding (n = 6 mice in each group). C,D, Food intake of STZ/HFD T2D mice (C) and db/db T2D mice (D) in indicated groups (n = 6 mice in each group). E,F, Water intake of STZ/HFD T2D mice (E) and db/db T2D mice (F) in indicated groups (n = 6 mice in each group). G,H, Blood glucose of STZ/HFD T2D mice (G) and db/db T2D mice (H) after 16 weeks of High-BCAA feeding (n = 6 mice in each group). I,J, Glucose tolerance were measured in STZ/HFD T2D mice (I) and db/db T2D mice (J) in indicated groups (n = 6 mice in each group). K,L, Insulin tolerance were measured in fasted STZ/HFD T2D mice (K) and db/db T2D mice (L) in indicated groups (n = 6 mice in each group). M, Western blot analysis of mTORC1 pathway and BCAA metabolic enzymes in heart tissue samples from STZ/HFD and db/db T2D mice treated with High-BCAA chow (n = 3 mice in each group). Data shown are expressed as mean ± SEM. $^{ns}P > 0.05$, $^{*}P < 0.05$, $^{***}P < 0.001$, using Student's *t*-test.

Table S1. Characteristics of Participants in Discovery group

Characteristics	Diabetics controls	DCM Patients	P Value
Number (n)	19	15	–
Age (years)	61.74±10.25	66.07±11.88	–
Male (%)	13 (68.42%)	5(33.33%)	–
BMI (kg/m^2)	24.41±4.00	23.76±3.30	0.6283 ns
Duration of diabetes (years)	8.74±4.04	14.74±4.24	0.006***
Antidiabetic drugs usage (%)	11 (57.89%)	11 (73.33%)	–
Heart rates (/min)	83.74±13.42	82.27±15.83	–
SBP (mm Hg)	128.37±14.68	125.47±13.61	0.5812 ns
DBP (mm Hg)	79.63±10.43	69.13±13.53	0.0220 *
Fasting blood glucose (mmol/L)	7.11±1.97	7.19±1.44	0.9003 ns
HbA1c (%)	8.06±2.36	7.68±1.43	0.6100 ns
TC (mmol/L)	3.95±1.16	4.03±0.72	0.8184 ns
Triglycerides(mmolL)	1.63±0.61	1.39±0.39	0.2299 ns
Creatinine (μmolL)	81.21±28.26	105.00±93.07	0.3291 ns
BUN (μmol/L)	7.63±2.43	9.17±3.83	0.1893 ns
Uric acid (μmol/L)	331.89±59.61	349.40±95.61	0.5429 ns
Cystatin c (mg/L)	1.19±0.43	1.70±0.83	0.0317 *
eGFR (mL/min/1.73m^2)	114.62±40.69	97.63±39.58	0.2573 ns
LV (mm)	42.79±3.25	56.20±3.73	<0.0001 ****
LA (mm)	36.21±6.34	45.47±7.49	0.0009 ***
RV (mm)	20.74±1.87	23.40±2.91	0.0046 **
RA Major (mm)	42.42±4.11	47.87±8.63	0.0294 *
RA Minor (mm)	33.21±3.52	39.00±8.85	0.0200 *
IVS (mm)	9.95±1.40	10.87±0.78	0.0385 *
LVPW (mm)	9.63±1.11	10.60±0.69	0.0085 **
E/e’	7.84±0.62	9.79±1.49	<0.0001 ****
LVEF (%)	61.16±1.56	52.67±4.79	<0.0001 ****
LVFS (%)	33.26±1.81	29.53±2.06	<0.0001 ****

Data are expressed as mean±SEM. The nonparametric two-tailed Student’s t-test was used to compare groups. Significance is indicated as *,$P < 0.05$;**,$P < 0.01$;***, $P < 0.001$;****, $P < 0.0001$.

Table S2. Characteristics of Participants in Validation group

Characteristics	①Healthy controls	②Diabetics Patients	③DCM Patients	①VS② P Value	②VS③ P Value
Number (n)	30	129	123	–	–
Age (years)	62.43±12.65	63.71±14.40	63.43±13.10	–	–
Male (%)	15 (50.00%)	65 (50.39%)	63 (51.22%)	–	–
BMI (kg/m^2)	23.66±2.35	25.47±5.35	25.55±5.07	0.0799 ns	0.9205 ns
Heart rates (/min)	73.63±8.38	81.59±12.90	86.52±16.24	–	–
SBP (mm Hg)	129.50±13.36	134.02±18.82	136.15±20.84	0.2438 ns	0.3948 ns
DBP (mm Hg)	73.40±11.81	78.01±11.32	77.41±13.33	0.0496*	0.7044 ns
Duration of diabetes (years)	–	9.09±5.06	13.80±5.49	–	<0.0001****
Antidiabetic drugs usage (%)	–	80 (62.06%)	83 (67.48%)	–	–
Fasting blood glucose (mmol/L)	4.93±0.67	7.66±2.51	8.37±3.45	<0.0001**	0.0613 ns
HbA1c (%)	5.05±0.52	8.14±1.68	8.07±1.74	＜0.0001**	0.7368 ns
TC (mmol/L)	4.52±1.08	4.35±1.19	4.34±1.20	0.4821 ns	0.9160 ns
Triglycerides (mmolL)	1.52±0.36	1.7±0.99	1.67±0.91	0.2534 ns	0.6192 ns
Creatinine (μmolL)	76.43±17.93	82.09±74.41	78.38±46.34	0.6819 ns	0.6390 ns
BUN (μmol/L)	7.76±7.60	7.36±3.79	6.83±2.78	0.6841 ns	0.2103 ns
Uric acid (μmol/L)	312.83±68.79	328.47±108.26	326.11±113.75	0.4535 ns	0.8667 ns
Cystatin c (mg/L)	0.99±0.27	1.20±0.62	1.32±0.63	0.0805 ns	0.1335 ns
eGFR (mL/min/1.73m^2)	110.87±39.26	120.11±47.42	132.07±64.68	0.3265 ns	0.0955 ns
LV (mm)	44.43±5.00	44.52±5.12	54.51±3.54	0.9341 ns	<0.0001****
LA (mm)	33.57±4.92	34.15±5.68	42.02±4.74	0.6084 ns	<0.0001****
RV (mm)	19.70±2.60	20.78±2.58	22.42±1.93	0.0431*	<0.0001****
RA Major (mm)	41.03±4.18	42.29±3.59	45.67±4.73	0.0999 ns	<0.0001****
RA Minor (mm)	32.73±3.23	33.40±5.32	37.02±4.07	0.5168 ns	<0.0001****
IVS (mm)	9.60±1.17	9.98±1.28	10.42±1.38	0.1372 ns	0.0098**
LVPW (mm)	9.20±1.05	9.55±0.94	10.30±1.38	0.0757 ns	<0.0001****
E/e’	7.43±0.80	7.79±0.83	9.94±1.62	0.0340*	<0.0001****
LVEF (%)	61.23±5.98	62.47±4.08	53.87±3.32	0.1813 ns	<0.0001****
LVFS (%)	32.87±1.20	33.53±2.98	29.56±2.63	0.2379 ns	<0.0001****

Data are expressed as mean±SEM. The nonparametric two-tailed Student’s t-test was used to compare groups. Significance is indicated as *,$P < 0.05$;**,$P < 0.01$;***, $P < 0.001$;****, $P < 0.0001$.

Table S3. Characteristics of Participants in Validation group

Characteristics	①Healthy controls	②Diabetics Patients	③DCM Patients	①VS② P Value	②VS③ P Value
Number (n)	30	30	30	–	–
Age (years)	58.23±19.43	62.66±10.04	65.57±11.60	–	–
Male (%)	18 (60.00%)	7 (76.67%)	16 (53.33%)	–	–
BMI (kg/m^2)	23.79±4.34	25.24±3.02	25.54±3.05	0.01441 ns	0.7194 ns
Heart rates (/min)	83.67±18.89	82.17±11.21	81.80±16.43	–	–
SBP (mm Hg)	129.70±16.12	136.30±20.90	141.97±21.60	0.1834 ns	0.3142 ns
DBP (mm Hg)	79.23±13.14	82.90±14.55	78.83±11.36	0.3182 ns	0.2404 ns
Duration of diabetes (years)	–	7.27±4.69	12.13±5.50	–	0.0006***
Antidiabetic drugs usage (%)	–	21 (70.00%)	26 (86.67%)	–	–
Fasting blood glucose (mmol/L)	5.58±1.14	8.40±3.08	8.21±2.38	<0.0001**	0.7951 ns
HbA1c (%)	6.40±0.87	7.86±1.38	8.76±1.81	＜0.0001**	0.0372*
TC (mmol/L)	4.25±0.75	4.39±1.00	4.46±1.05	0.5658 ns	0.7797 ns
Triglycerides (mmolL)	1.40±0.82	1.72±0.66	1.76±0.91	0.1085 ns	0.8187 ns
Creatinine (μmolL)	72.87±17.55	78.40±31.50	74.87±20.71	0.4120 ns	0.6157 ns
BUN (μmol/L)	6.72±1.83	7.51±3.15	7.77±2.01	0.2428 ns	0.7192 ns
Uric acid (μmol/L)	325.23±104.81	351.83±132.10	332.73±127.82	0.3991 ns	0.5779 ns
Cystatin c (mg/L)	1.16±0.28	1.26±0.47	1.28±0.44	0.3523 ns	0.8628 ns
eGFR (mL/min/1.73m^2)	121.07±32.10	121.63±38.13	112.65±30.19	0.9520 ns	0.3242 ns
LV (mm)	44.77±3.71	44.43±5.21	52.17±4.55	0.7801 ns	<0.0001****
LA (mm)	33.77±5.76	35.57±3.81	42.47±4.10	0.1658 ns	<0.0001****
RV (mm)	20.07±2.22	20.70±1.44	22.03±2.20	0.2027 ns	0.0083**
RA Major (mm)	42.80±4.22	42.40±3.12	46.60±3.02	0.6829 ns	<0.0001****
RA Minor (mm)	33.07±4.22	33.60±4.61	36.77±4.30	0.6178 ns	<0.0001****
IVS (mm)	9.87±1.28	10.17±1.32	11.23±1.54	0.3837 ns	0.0098**
LVPW (mm)	9.37±1.14	9.60±0.99	10.57±1.48	0.4079 ns	<0.0001****
E/e’	7.38±0.63	7.55±0.58	10.04±1.46	0.3011 ns	<0.0001****
LVEF (%)	61.50±5.18	62.40±1.80	54.30±7.67	0.3806 ns	<0.0001****
LVFS (%)	33.63±2.07	33.83±1.61	29.20±3.75	0.6834 ns	<0.0001****

Data are expressed as mean±SEM. The nonparametric two-tailed Student’s t-test was used to compare groups. Significance is indicated as *,$P < 0.05$;**,$P < 0.01$;***, $P < 0.001$;****, $P < 0.0001$.

Table S4. Amino Acids Rodent Diet and Same Halved or Double BCAA

**Diet#**	**FB-A10021B** Control		**FB-A10022** Halved BCAA		**FB-A10022** Double BCAA	
	gm%	*kcal%*	gm%	*kcal%*	gm%	*kcal%*
Protein	17.00	*17.56*	15.60	*16.12*	19.80	*20.45*
Carbohydrate	68.55	*70.82*	69.95	*72.26*	65.75	*67.92*
Fat	5.00	*11.62*	5.00	*11.62*	5.00	*11.62*
Total	90.55	*100.00*	90.55	*100.00*	90.55	*100.00*
Ingredient(gm)	gm	*kcal*	gm	*kcal*	gm	*kcal*
L-Arginine	10	*40*	10	*40*	10	*40*
L-Histidine-HCl-H_2O	6	*24*	6	*24*	6	*24*
L-Isoleucine	8	*32*	4	*16*	16	*64*
L-Leucine	12	*48*	6	*24*	24	*96*
L-Valine	8	*32*	4	*16*	16	*64*
L-Tyrosine	4	*16*	4	*16*	4	*16*
L-Lysine-HCl	14	*56*	14	*56*	14	*56*
L-Methionine	6	*24*	6	*24*	6	*24*
L-Phenylalanine	8	*32*	8	*32*	8	*32*
L-Threonine	8	*32*	8	*32*	8	*32*
L-Tryptophan	2	*8*	2	*8*	2	*8*
L-Alanine	10	*40*	10	*40*	10	*40*
L-Asparagine-H_2O	5	*20*	5	*20*	5	*20*
L-Aspartate	10	*40*	10	*40*	10	*40*
L-Cystine	4	*16*	4	*16*	4	*16*
L-Glutamic Acid	30	*120*	30	*120*	30	*120*
L-Glutamine	5	*20*	5	*20*	5	*20*
Glycine	10	*40*	10	*40*	10	*40*
L-Proline	5	*20*	5	*20*	5	*20*
L-Serine	5	*20*	5	*20*	5	*20*
Corn Starch	550.5	*2202*	564.5	*2258*	522.5	*2090*
Maltodextrin	125	*500*	125	*500*	125	*500*
Cellulose	50	*0*	50	*0*	50	*0*
Corn Oil	50	*450*	50	*450*	50	*450*
Mineral Mix	35	*0*	35	*0*	35	*0*
Sodium Bicarbonate	7.5	*0*	7.5	*0*	7.5	*0*
Vitamin Mix	10	*40*	10	*40*	10	*40*
Choline Bitrartrate	2	*0*	2	*0*	2	*0*

Yellow Dye	0	*0*	0	*0*	0.025	*0*
Blue Dye	0.05	*0*	0.025	*0*	0.025	*0*
Red Dye	0	*0*	0.025	*0*	0	*0*
Total	1000.05	*3872*	1000.05	*3872*	1000.05	*3872*
